# Trypsin Digestion Conditions of Human Plasma for Observation of Peptides and Proteins from Tandem Mass Spectrometry

**DOI:** 10.1021/acsomega.4c03955

**Published:** 2024-09-24

**Authors:** Zhuo Zhen Chen, Jaimie Dufresne, Peter Bowden, Ming Miao, John G. Marshall

**Affiliations:** Research Analytical Biochemistry Laboratory, Department of Chemistry and Biology, Toronto Metropolitan University, Toronto M5B 2K3, Canada

## Abstract

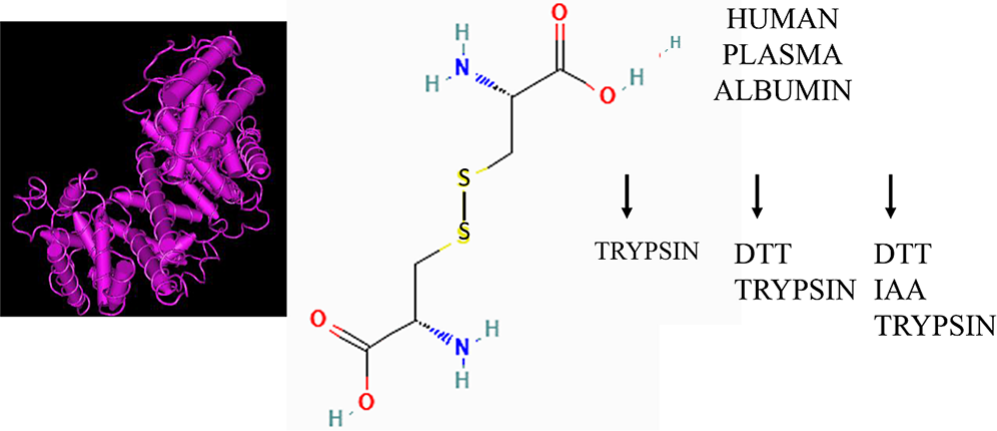

Previous meta-analysis indicated that plasma or serum proteome groups using various experimental conditions detected different peptides from the same plasma proteins, which is strong evidence for the veracity of blood fluid LC-ESI-MS/MS but also evidences that the trypsin digestion step is a key source of variation in plasma proteomics. Agreement between different digestion conditions and MS/MS algorithms may serve as an independent confirmation of the validity of the LC-ESI-MS/MS analysis of plasma peptides. Plasma contains a high percentage of albumin held together by multiple disulfide bonds; hence, reduction and/or alkylation may greatly enhance the digestion efficiency of albumin. Plasma proteins were precipitated in 90% acetonitrile, collected over quaternary amine resin, and eluted in NaCl prior to digestion treatments. To determine the effect of trypsin digestion methods, the plasma proteins were digested in 600 mM urea and 5% acetonitrile with trypsin alone, or reduced with 2 mM DTT followed by trypsin, or DTT followed by 15 mM iodoacetamide and then trypsin. The resulting peptides were analyzed by LC-ESI-MS/MS with a linear quadrupole ion trap (LIT). The MS/MS spectra were directly fit to peptides by the X!TANDEM and SEQUEST algorithms. Blank noise injections served as the analytical control, and 30 million random MS/MS served as the statistical control. Digesting human plasma with DTT reduction, or reduction and alkylation, resulted in a dramatic increase in the number and observation frequency of albumin peptides. In contrast, digestion with trypsin alone suppressed the observation of albumin, and instead, many low abundance plasma and cellular proteins showed higher observation frequency. Digestion with trypsin alone increased the observation frequency of APOC1, ACAN, ATRN, CPB2, GP2, GPX3, HBA1, PAPD5, PKD1, and many cellular proteins. After correction against noise and random controls, SEQUEST showed good agreement with the true positive plasma proteins identified by X!TANDEM and resulted in an *R*-squared of 0.5238 with an *F*-statistic of 10,930 on 9,935 protein gene symbols with a *p*-value < 2.2e–16. Digestion of plasma with trypsin alone avoids the complete digestion of albumin and permits the enhanced detection of some other cellular proteins from plasma. Different digestion approaches were complimentary and together resulted in a more comprehensive plasma proteome. The protein FDR *q*-values, the modest effect of background and Monte Carlo correction, and the significant STRING analysis were all consistent with the high fidelity of the rigorous X!TANDEM algorithm. In contrast, SEQUEST required significant correction against noise and statistical controls and selection of high cross correlation (XCorr) scores to show good agreement with X!TANDEM. There was qualitative and quantitative agreement between plasma proteins digested without alkylation from the orbital ion trap (OIT) versus the LIT instrument that showed highly significant regression against the X!TANDEM OIT monoisotopic results, those from heavy isotopes and other masses from X!TANDEM, and with those from MaxQuant. There was significant qualitative and quantitative agreement between the complementary digestion conditions consistent with the good fidelity of plasma analysis by LC-ESI-MS/MS with a sensitive linear ion trap.

## Introduction

The trypsin digestion step was apparently an important point of variation in the LC-ESI-MS/MS analysis of blood proteins.^1^ A previous meta-analysis of peptides and proteins from blood fluids reported by multiple groups indicated that the independent laboratories often reported different peptide species from the same set of at least 12,000 proteins, which is powerful evidence for the veracity of LC-ESI-MS/MS but also suggests that the process may be sensitive to digestion conditions.^1−5^ The strong level of agreement between peptides and proteins from different methods provides powerful statistical evidence for the veracity of LC-ESI-MS/MS.^6,7^ Plasma proteins consist of a large proportion (∼50%) of albumin.^8,9^ Albumin has a compact globular structure supported by 17 disulfide bonds^10^ and has an acidic isoelectric point^9^ that binds to anion exchange resin.^11^ Similarly, the abundant immunoglobulins are held in their structures by up to 12 disulfide bonds^12^ and may be retained by quaternary amine (QA), that is, strong anion exchange (SAX) chromatography.^13,14^ There have been many attempts to remove albumin, IgG, and other abundant proteins from plasma by chromatography prior to digestion.^15^ Reduction and alkylation prior to tryptic digestion identified 490 high confidence proteins.^16,17^ In contrast, ∼700 major serum proteins were identified from high scoring, fully tryptic peptides by digestion with trypsin alone in the absence of DTT and alkylation,^18^ and that figure was subsequently confirmed.^19^ Digestion in tris pH 8.8 in 600 mM Urea and 5% ACN with trypsin alone overnight prior to reduction in 2 mM DTT and brief redigestion with trypsin in the absence of alkylation yielded 4,396 distinct protein gene symbols from fully tryptic peptides by the rigorous X!TANDEM algorithm from relatively insensitive microflow electrospray to Paul ion trap,^11^ and that number has now been independently verified by the Human Proteome Organization (HUPO) that showed 4,395 proteins.^20^ It may be possible to increase the observation frequency of some low abundance plasma proteins by avoiding the digestion of albumin under different tryptic digestion conditions. Albumin must be reduced and/or alkylated to achieve complete digestion.^21^ In addition to efficiently digesting albumin, reduction and treatment by iodoacetamide might also result in unexpected structural modifications of peptides.^22^ Proteomic analysis of serum or plasma and other samples has been successfully performed using trypsin without reduction and alkylation^18,23^ or by digestion in urea with trypsin alone overnight ≥ 16 h, followed by reduction in DTT and a second brief digestion.^11,24−29^ Here, human EDTA plasma was precipitated in acetonitrile,^30^ collected over quaternary amine resin,^11^ tested for protein content,^31^ and dissolved in 600 mM urea with 5% acetonitrile. The effect of digestion conditions with trypsin alone^18^ was compared to DTT followed by trypsin and DTT followed by alkylation with iodoacetamide (15 mM) prior to trypsin digestion. The digests were analyzed by LC-ESI-MS/MS, and the fragmentation spectra fit to fully tryptic peptides by the X!TANDEM algorithm, which was previously demonstrated to have a low type I (false positive) error rate by Monte Carlo statistical control,^32^ versus the sensitive SEQUEST algorithm. X!TANDEM is a highly rigorous algorithm that generates a *p*-value using the fundamental statistical principle of goodness of fit directly from the match of MS/MS spectra to those predicted for peptides.^32^ SEQUEST is a highly sensitive algorithm that directly generates an X-Corr value from the fundamental statistical metric of cross correlation directly from the fit of MS/MS spectra to those predicted for peptides.^33^ Thus, peptides and proteins can be accurately identified and quantified by fit of the MS/MS spectra (±0.5 Da) alone in the absence of accurate peptide precursor mass values.^26,27,32,34,35^ Agreement between the X!TANDEM and SEQUEST algorithms that operate on independent, fundamental statistical approaches has previously been used to demonstrate the validity of LC-ESI-MS/MS results.^6,7^ The goodness of fit (X!TANDEM) and cross correlation (SEQUEST) algorithms may be used directly on the fragment MS/MS spectra and are the only two fragment fitting algorithms that have been validated against the bedrock statistical control of the Monte Carlo simulation using millions of random MS/MS spectra.^7,26,27,36^ The observation that large molecules like peptides may ionize into the gas phase with heavy isotopes and hydrogen rearrangements means that in nature peptides may ionize with a wide mass distribution.^37,38^ The fit of MS/MS spectra to peptide sequences at a range of peptide mass may be used to explain all MS/MS spectra without arbitrary empirical or heuristic approaches.^7,24,25,28,29^ The fit of peptides using well-established classical statistical methods like goodness of fit, cross correlation, and Monte Carlo correction is the simplest model that explains all MS/MS spectra and leads to testable hypotheses.^7,26,27,36^ It has been previously demonstrated that simple ion traps agree with high-resolution hybrid mass spectrometers, though the lower resolution ion traps are more sensitive.^18,24,39−41^ Specifically, the linear quadrupole ion trap identifies the same plasma proteins as the orbital trap from MS/MS^24^ but is about 10 times more sensitive in terms of signal-to-noise cut off at low ion intensity values.^41^ Here, the X!TANDEM and SEQUEST algorithms showed that the observation frequency of peptides from albumin was dramatically increased by reduction and alkylation. In contrast, the level of detection of many less abundant plasma proteins was increased by digestion with trypsin alone. Different digestion methods were complementary, and the sum of the three methods provided the largest human plasma proteome recorded to date that shows excellent agreement with the estimated size of the serum proteome and the combined results of many laboratories.^1,2^

## Results

**Figure 1:**
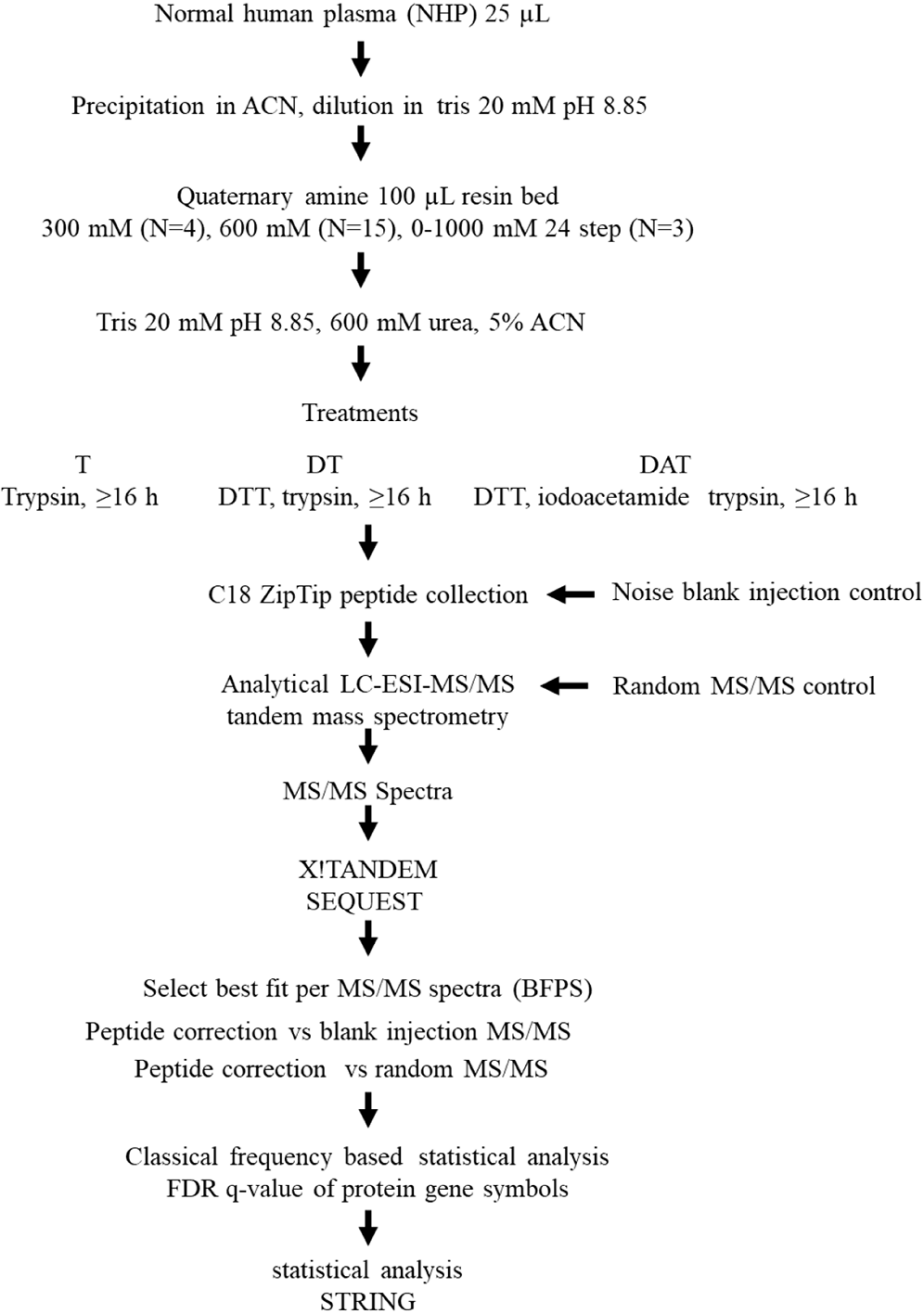
Separation of intact plasma proteins by quaternary amine (SCX) resin prior to comparison of tryptic digestion without reduction (T), with DTT reduction and trypsin digestion (DT), and with DTT reduction, (IAA) alkylation, and trypsin (DAT) followed by LC-ESI-MS/MS and statistical analysis. The results of 29 nano LC-ESI-MS/MS blank (mock) injections served as a baseline analytical control. In addition, 30,000,000 random MS/MS spectra served as statistical controls. The peptide and protein *p*-values were computed by X!TANDEM and corrected by the FDR method of Benjamini and Hochberg. Each of the three treatments T, DT, and DAT had three separate experiments one with a single step of 300 mM NaCl (*N* = 4), one with a single step of 600 mM NaCl (*N* = 15), and one with 24 steps from 50 to 1000 mM NaCl (*N* = 3), resulting in 91 LC-ESI-MS/MS experiments per treatment.

Here, the effect of trypsin digestion conditions on the plasma proteome was investigated as determined by the X!TANDEM and SEQUEST algorithms corrected against the analytical control of blank LC-ESI-MS/MS injections and the statistical control of 30,000,000 random MS/MS spectra (Figure 1). Precipitation of plasma in acetonitrile, collection over disposable quaternary amine (QA) micro chromatography prior to digestion with C18 ZipTip prior to dilution and manual injection for analytical LC-ESI-MS/MS in mass spectrometry grade solvents and water was previously shown to be a highly effective means to identify plasma proteins.^11,24,25,42^ The effect of digestion in tris pH 8.85 with 600 mM urea and 5% acetonitrile using (1) trypsin, or (2) reduction in DTT followed by trypsin, or (3) reduction in DTT and alkylation with iodoacetamide followed by trypsin was compared on human plasma samples. The experiments produced 5,410,083 MS/MS spectra from 273 LC-ESI-MS/MS experiments (Table 1). The results indicated that the human peptides observed by LC-ESI-MS/MS and fit by the X!TANDEM and SEQUEST were very sensitive to digestion conditions with trypsin alone, resulting in the most efficient collection of peptide observations from most proteins. Selecting the single best fit per spectra (BFPS) match with correction against analytical and statistical controls resulted in 143,062 accepted peptides from X!TANDEM (2.6% MS/MS) and resulted in 418,768 peptides from SEQUEST (XCorr ≥ 2, 7.7% of MS/MS) with ≥ 12,000 protein gene symbols (Table 1). Digestion of blood proteins by different methods results in a large variation in the observation frequency of the proteins detected. The different digestion methods had a marked effect on the observation frequency of many proteins, but digestion in trypsin alone reduced observation of albumin. The digestion methods were complimentary, and the combination of the digestion methods resulted in a larger plasma proteome with at least 9,472 protein gene symbols with 3 peptides from rigorous X!TANDEM with an FDR *q*-value ≤ 0.01 were shown. A much larger number of proteins were identified by SEQUEST but required significant correction against random MS/MS spectra at the level of peptide sequences. The SEQUEST algorithm typically provided a higher observation frequency value for the high confidence set of proteins identified by X!TANDEM.^7,24,25,43^

**Table 1:** An SQL Server Summary of the Effect of Trypsin Digestion Treatments on Peptides and Protein Accessions Matched by LC-ESI-MS/MS^a^^b^^c^^d^^e^^f^^g^^h^^i^

X!TANDEM							
Digestion treatment	MS runs	MS/MS count	Total matched distinct spectra	BFPS counts noise corrected	Monte Carlo random MS/MS corrected	Peptide *p*-value < 0.1	Gene symbol *N* ≥ 1
DTT, IAA, and trypsin	91	1,829,266	65,894	65,182	54,555	54,555	11,390
DTT and trypsin	91	1,908,258	39,704	39,358	32,360	32,360	9,265
Trypsin alone	91	1,672,559	65,848	65,269	56,147	56,147	12,137
Total	273	5,410,083	171,446	169,809	143,062	143,062	17,978

SEQUEST							
Digestion treatment	MS runs	MS/MS count	Total matched distinct spectra	BFPS counts noise	Monte Carlo random subtracted	Peptide XCorr ≥ 2	Gene symbol *N* ≥ 1
DTT, IAA, and trypsin	91	1,829,266	1,827,730	1,490,959	1,092,199	133,719	13,778
DTT and trypsin	91	1,908,258	1,905,926	1,680,738	1,336,428	176,623	14,833
Trypsin aslone	91	1,672,559	1,655,268	1,320,282	834,637	108,426	17,181
Total	273	5,410,083	5,388,924	4,491,979	3,263,264	418,768	20,703

a The trypsin digestion buffer was 20 mM tris pH 8.85 with 600 mM urea and 5% acetonitrile.

b Treatments: trypsin alone (T), DTT reduction followed by trypsin (DT), DTT reduction IAA alkylation, and trypsin (DAT).

c Digestion for ≤ 16 h (overnight) with 1/100 ug of trypsin/ug of protein.

d The best fit per MS/MS spectra (BFPS) was selected in the SQL Server.

e Any peptide sequences also observed in 29 blank (noise) injection LC-ESI-MS/MS experiments were discarded.

f Any peptide with an observation frequency that was not resolved from those of 30,000,000 random MS/MS spectra was discarded.

g Peptides with X!TANDEM *p*-values ≤ 0.1 were accepted.

h Peptides with a SEQUEST XCorr value ≥ 2 were accepted.

i Gene symbol values prior to computation of protein FDR *q*-values in the R statistical system are shown.

### Digestion of Albumin and Other Proteins

The commonly used method of reduction and alkylation to achieve complete digestion and peptide coverage was developed using the globular protein albumin that has 17 disulfide linkages and hence is somewhat resistant to tryptic digestion.^21,44^ Trypsin alone showed 3,787/12,946 (X!TANDEM/SEQUEST) peptide counts from albumin, while DTT reduction prior to digestion showed 4,700/106,989 peptide counts, and reduction and alkylation prior to digestion resulted in 12,402/83,098 peptide observations from albumin. The results indicated that trypsin does not efficiently digest albumin without reduction or alkylation and results in only about 30% of the albumin observations from reduction or alkylation together. Thus, avoiding reduction and alkylation permitted the LC-ESI-MS/MS system to devote much less effort to sampling peptides from albumin that increased the observation frequency of many plasma and (Table S1) and cellular proteins (Table S2).

### Peptide Correlation to Protein Accessions

#### X!TANDEM

**Figure 2:**
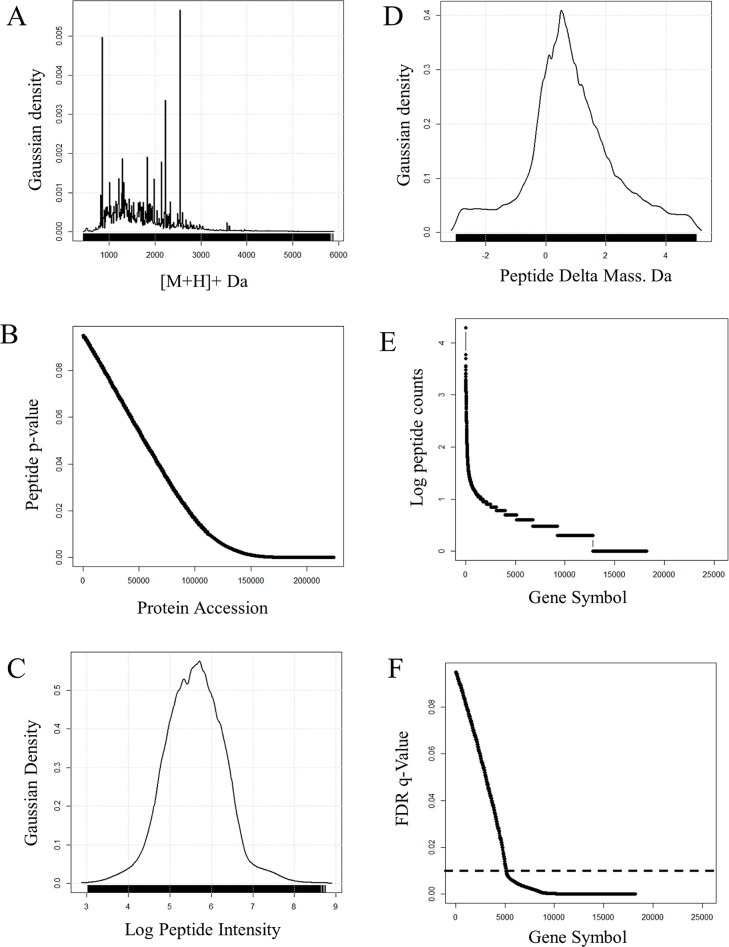
Statistical analysis of the sum of the LIT digestion data computed and graphically analyzed with the R statistical system. The human plasma digests were computed at the level of MS/MS spectra, peptides, protein accession number, and protein gene symbols from the sum of X!TANDEM from all three digestion conditions. (A) The MH distribution of peptides in daltons. (B) The peptide *p*-value distribution of all peptides matched to protein accessions. (C) The peptide log precursor intensity distribution. (D) The peptide delta mass distribution (observed–expected mass) in daltons (Da). (E) The distribution log peptide counts over protein gene symbols. (F) The FDR *q*-value of protein gene symbols computed in the R statistical system. The dotted line shows ≥ 12,000 protein gene symbols below the *q* ≤ 0.01 cutoff.

Analysis at the level of peptides, protein accessions, and gene symbols shows that 56% of peptides from X!TANDEM were in z = 2+ charge state and the remainder in z = 3+ charge. The peptide masses from X!TANDEM were distributed from about 500 MH to 6,000 MH but were mostly from 1,000 to 3,000 MH where isotopes are predominant (Figure 2A). The *p*-values from the fit of MS/MS spectra (±0.5 Da) of individual peptides ranged from *p* < 1e–1 to *p* < 1e–16 (machine 0), and X!TANDEM successfully matched the 5,410,083 MS/MS to 225,068 peptides (Figure 2B). The log precursor intensity values were Gaussian from e4 to e7 arbitrary counts (Figure 2C) where the signal-to-noise cutoff for the LTQ is about e3 counts.^26,27,41,45,46^ The delta mass values were distributed from −2 to +4 Da but were concentrated in the range of 0, +1, and +2 Da, consistent with the presence of heavy isotopes^37,38^ (Figure 2D). The MS/MS to peptide mapping in SQL selected the single best fit per spectra (BFPS), and those peptides typically matched to one but up to tens of accessions as computed in the SQL Server database. In turn, the protein accessions typically mapped to a single gene symbol but were sometimes associated with multiple gene symbols. Thus, the SQL Server database was able to store and relate all MS and MS/MS spectra, peptide matches, protein accessions, and gene symbols for classical statistical analysis in R.^36^ More than 9,000 protein gene symbols (peptide *N* ≥ 3), or where none available loci or accessions, were observed by X!TANDEM after correction against blank injections and random MS/MS (Figure 2E), and more than 12,000 proteins show an FDR of ≤ 1% (FDR *q* ≤ 0.01) (Figure 2F).

#### SEQUEST

After corrections, about 37% of peptides from SEQUEST were z = 2+ and the remainder were z = 3+ charge state. SEQUEST fit peptides over a wide range of MH values focused from 500 to 3,000 Da (Figure S1A). SEQUEST showed a high redundancy and attempted to match the 5,410,083 MS/MS spectra to over 60 million peptides (Figure S1B inset) with about half a million XCorr values greater than 2 that was the major inflection point of the data (see arrow in Figure S1B).

The log precursor intensity values were Gaussian and ranged from e4 to e7 arbitrary detector counts (Figure S1C). The delta mass distribution of the SEQUEST data was focused on the 0, +1, or +2 Da isotope range after correction (Figure S1D) but showed a random distribution prior to correction (Figure S1D inset). The MS/MS to peptide mapping in SQL to select the single best fit per spectra (BFPS) resulted in a single peptide sequence per MS/MS spectra (Figure S1E inset), and those peptides typically matched to one but up to tens of accessions as computed in the SQL Server database (Figure S1E). In turn, the protein accessions typically mapped to a single gene symbol but were sometimes associated with many entries where annotation was unavailable (Figure S1F).

### Missed Cleavages and CAM Modification of Cysteine from X!TANDEM

**Figure 3:**
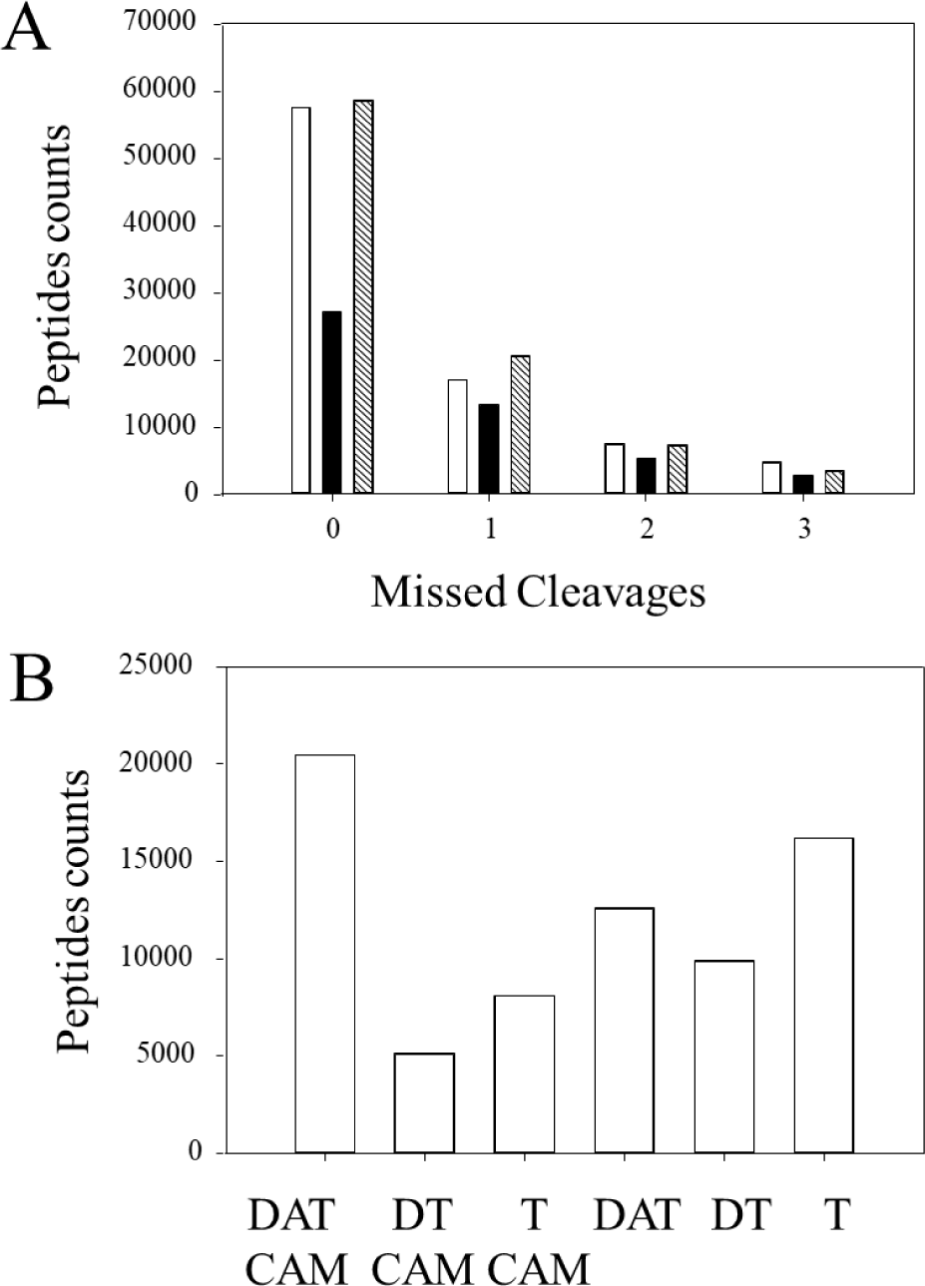
Effect of digestion methods on missed cleavages and glycine modification (i.e., carbamidomethyl modification, CAM) of cysteine (57.02 Da) from X!TANDEM. The comparison of digestion treatments from the LIT: trypsin = T, DTT trypsin = DT, and DTT IAA trypsin = DAT. (A) The missed cleavages per peptide from DAT (white), DT (black), and T (hatched) from X!TANDEM. (B) The effect of digestion treatments on carbamidomethyl modification (CAM) versus no modification at cysteine from X!TANDEM.

Different digestion methods had little impact on the proportion of missed cleavages where the large majority of peptides had 0 missed cleavage and minority had 1 missed cleavage, 2 missed cleavages were rare, and 3 or more approached negligible levels (Figure 3A). The effect of digestion treatments on carbamidomethyl modification at cysteine (CAM) showed reduction- and alkylation-modified cysteine on ≥ 20,000 distinct peptides, which is about 4-fold greater than DTT (Figure 3B). The largest number of unmodified peptides was from trypsin alone. A table of high and low abundance plasma proteins with and without cysteine modification is provided in Table S3. There are a large number of modifications to cysteine that occur in plasma proteins including glycine or carboxymethyl modifications that may have physiological relevance.^47,48^ The b and y fragment ion series of cysteine peptides like QNCELFEQLGEYK confirmed the precise localization of the 57 Da modification to cysteine from the b and y ion series with the X!TANDEM algorithm. The peptide QNCELFEQLGEYK with the 57 Da cysteine modification reflected in the precursor and fragments was observed 426 times from DAT, 10 times from DT, and 30 times from the T treatment. The number of CAM modifications observed is the highest in IAA treatment, as expected. The observed number is lowest in DTT treatment, where cysteine bridge amino acids would be exposed to trypsin without modification. As expected, the number was somewhat greater in trypsin alone where cysteines that were not participating in bridges and thus might be exposed to derivatization in vivo^47,48^ and could be sampled by trypsin.

### Comparison of X!TANDEM versus SEQUEST Protein Gene Symbols

The X!TANDEM algorithm generates a *p*-value from the goodness of fit of observed versus predicted MS/MS spectra, where the signal-to-noise ratio is high. Digestion with trypsin alone generated 6,500 protein gene symbols from the best fit per MS/MS spectra (BFPS) with an FDR *q*-values less than 1% (*q* ≤ 0.01) (Figure S2A). Reduction with DTT prior to trypsin produced 4,534 significant protein gene symbols (Figure S2B). Reduction with DTT and alkylation with iodoacetamide prior to trypsin resulted in 6,214 significant protein gene symbols (Figure S2C). The combination of methods yielded a total of 12,808 proteins with at least 2 peptides and 13,087 protein gene symbols with an FDR *q*-values less than 1% (*q* ≤ 0.01) (Figure S2D). SEQUEST analysis of plasma digested with trypsin alone (T) identified more peptides from more proteins and gene symbols XCorr ≥ 2 than reduction and/or alkylation (Figure S2E–G). After selecting the best fit per spectra (BFPS) peptides and correcting against noise and random MS/MS spectra together with imposing an XCorr cutoff score ≥ 2, then SEQUEST fit *N* ≥ 3 peptides from ≥ 15,000 protein gene symbols from the sum of the three enzyme digestion methods (Figure S2H).

### Agreement of Independent X!TANDEM versus SEQUEST Algorithms

**Figure 4:**
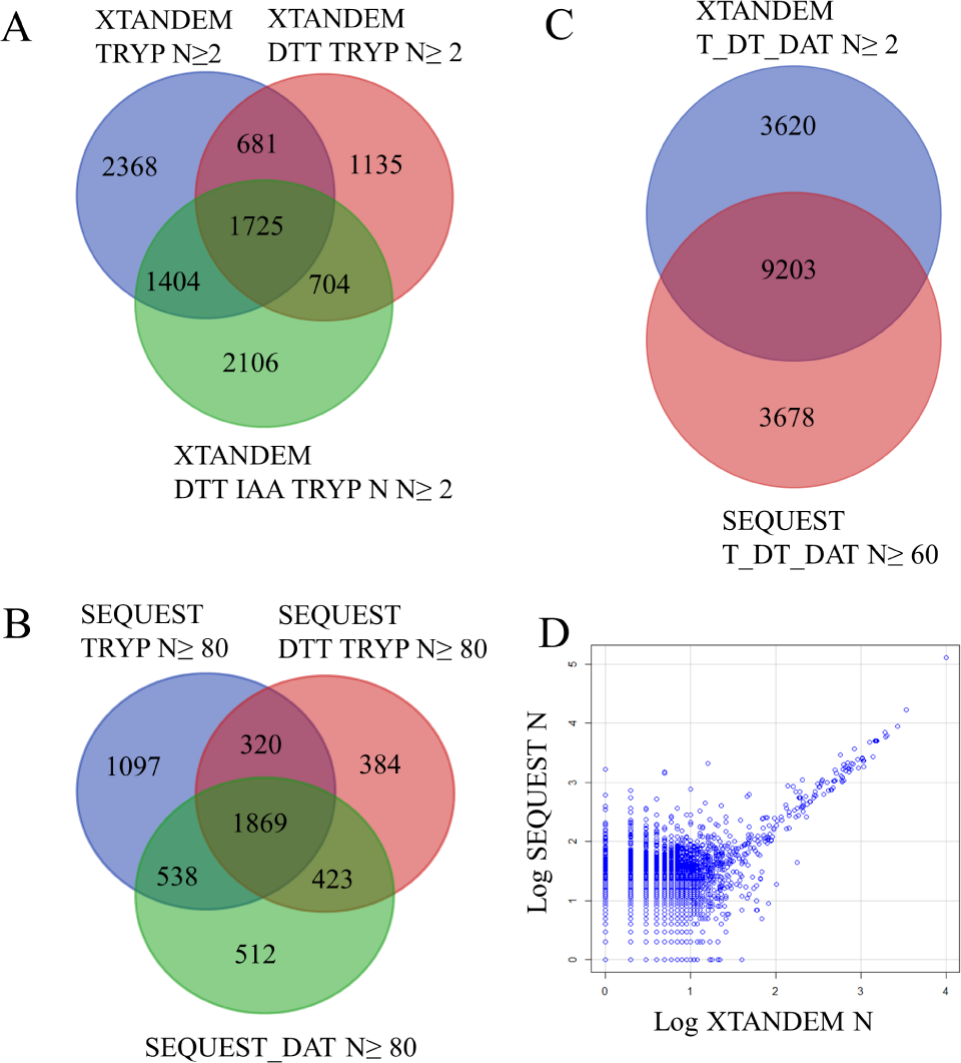
Qualitative and quantitative agreement between digestion treatments (T, DT, and DAT) from the LIT instrument shown by Venn diagrams and scatter plot at the level of protein gene symbols. The effect of trypsin alone, DTT, and alkylation on the number of gene symbols observed by X!TANDEM as the sum of the independent tryptic (TRYP) versus phosphotryptic (STYP) peptides. (A) The Venn diagram of protein gene symbols with at least 2 (*N* ≥ 2) peptide observations. (B) The Venn diagram of SEQUEST protein gene symbols with at least 80 peptide observations (*N* ≥ 80). (C) The Venn diagram of the sum of digestion treatments (T_DT_DAT) by X!TANDEM (*N* ≥ 2) versus SEQUEST (*N* ≥ 60). (D) The scatter plot of corrected X!TANDEM versus corrected SEQUEST XCorr ≥ 2 peptide counts per gene symbol [*R*^2^ of 0.5239, an *F* value of 10,930 on 9,935 DF, and a *p*-value near machine zero (2.2e–16)]. The BFPS peptide counts per protein gene symbol after correction against noise MS/MS analytical and random MS/MS statistical controls are shown. The log peptide counts per gene symbol (N) after correction against noise and random MS/MS spectra are plotted.

There was good agreement on the number and observation frequency of plasma proteins between SEQUEST and X!TANDEM. Each MS/MS spectrum in the study was identified by a unique identification number (spectral_ID#) that was assigned to the best fit per spectra by X!TANDEM versus SEQUEST where a complex key query was employed to prevent any MS/MS spectra from being assigned to more than one peptide by X!TANDEM or SEQUEST to remove errors from redundancy. Venn diagram analysis of stringent X!TANDEM proteins with at least 2 peptides and thus ≤ 1% type I error (*p* ≤ 0.01) showed that direct digestion with trypsin had 2,368 specific proteins that were more productive than reduction and alkylation, which showed 2,106 specific proteins (Figure 4A). Venn diagrams of SEQUEST showed 1,097 proteins with *N* ≥ 80 BFPS peptide specific to direct trypsin digestion, while reduction and alkylation showed 512 proteins with *N* ≥ 80 BFPS peptides (Figure 4B). Comparing the top 12,000 protein gene symbols between X!TANDEM and SEQUEST showed about 9,000 protein (75%) agreement, which is a remarkable overlap for such a large set of proteins (Figure 4C). After correction against noise and random MS/MS spectra, regression of SEQUEST results in the set of true positive X!TANDEM protein gene symbols (X!TANDEM *n* ≥ 3 peptides) and in a strong quantitative relationship for the set shared protein gene symbols with an *R*^2^ of 0.5239, an *F* value of 10,930 on 9,935 DF, and a *p*-value near machine zero (2.2e–16). Regression analysis of the two algorithms showed quantitative agreement that is a statistically powerful estimate of the veracity of LC-ESI-MS/MS (Figure 4D). After noise and random MS/MS correction, SEQUEST peptides with XCorr values ≥ 2 showed 9,551 protein gene symbols in agreement with X!TANDEM *n* ≥ 2 (see Supporting Information).

### Agreement on the Plasma Proteome

The STRING algorithm was employed to graphically present the agreement between the top 100 protein gene symbols from X!TANDEM (Figure S3A) and the corrected SEQUEST where XCorr was ≥ 2 (Figure S3B). X!TANDEM detected a network of proteins from multiple apolipoproteins, hemopexin, haptoglobin, complements, transferrin FGG, FGA FGB, VTN, and hemoglobins in agreement with SEQUEST that also showed the presence of some HLA (MHC) proteins and granzyme A (GXMA).

The overlap set of X!TANDEM and SEQUEST XCorr ≥ 2 after corrections against noise and random MS/MS spectra is presented in Supporting Information with the average XCorr and *p*-values and FDR corrected *q*-values using the method of Benjamini and Hochberg.^49^

### Orbital Ion Trap (OIT)

**Figure 5:**
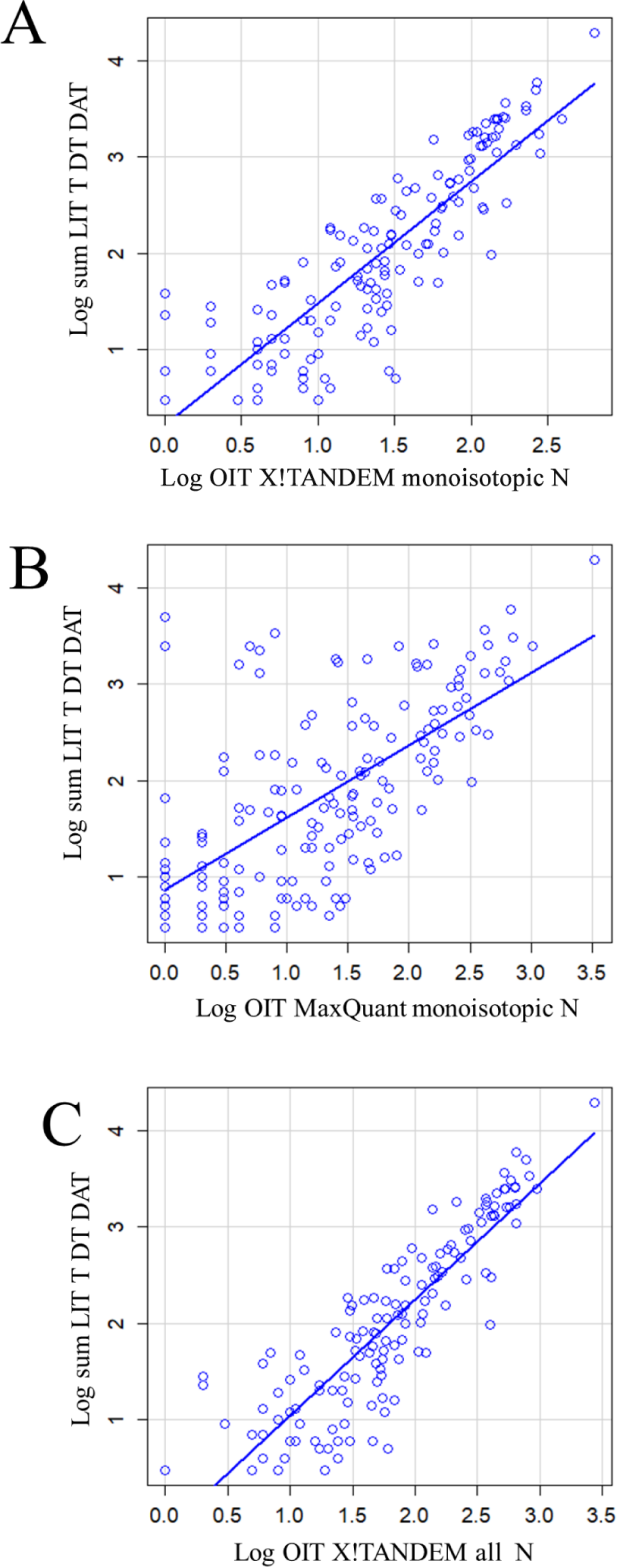
Quantitative agreement of the plasma trypsin digestion between the LTQ XL (LIT) data and the orbitrap (OIT) instruments was performed by X!TANDEM and MaxQuant. (A) Scatter plot of the relation between the sum of the LIT digestion results versus the OIT from X!TANDEM monoisotopic results only (residual standard error: 0.4018 on 8,299 degrees of freedom, multiple *R*-squared: 0.328, *F*-statistic: 4,050 on 1 and 8,299 DF, and *p*-value: <2.2e–16). (B) Scatter plot of the relation between the sum of the digestion methods from the LIT versus the versus orbital trap result from MaxQuant (residual standard error: 0.7687 on 203 degrees of freedom, multiple *R*-squared: 0.5046, *F*-statistic: 206.8 on 1 and 203 DF, and *p*-value: <2.2e–16). (C) Scatter plot of the relation between the sum of the LIT versus the orbital trap from all X!TANDEM delta mass values (residual standard error: 0.4018 on 8,299 degrees of freedom, multiple *R*-squared: 0.328, adjusted *R*-squared: 0.3279, *F*-statistic: 4,050 on 1 and 8,299 DF, and *p*-value: <2.2e–16).

The digestion of plasma proteins was repeated without alkylation by IAA and analyzed by an orbital ion trap (OIT) instrument for comparison with the results of the linear ion trap (LIT). The OIT results were matched to peptides using the rigorous X!TANDEM goodness of fit of MS/MS spectra versus the MaxQuant heuristic algorithm that only fits monoisotopic peptides. There was excellent and quantitative agreement between the sum of the digestion methods from the LIT, plotted against OIT monoisotopic peptides from X!TANDEM (Figure 5A), against the OIT monoisotopic peptides from MaxQuant (Figure 5B), or against all the isotopic and other peptides from X!TANDEM (Figure 5C) that all showed highly significant (∼*p* < 2e-16) quantitative agreement by regression. The goodness of fit of MS/MS spectra to monoisotopic peptides from X!TANDEM was apparently more rigorous than MaxQuant, which uses a heuristic score based heavily on precursor mass (Table S4). For example, X!TANDEM identified only 642 monoisotopic (±0.1 Da) peptides from albumin from the goodness of fit of MS/MS spectra, while MaxQuant identified 3,336 albumin peptides from the monoisotopic mass. However, in this small experiment, we estimated that MaxQuant suffered from a 14% false positive rate from the redundant use of MS/MS spectra that is much larger than 1% error.

## Discussion

The aim of this study was to compare trypsin digestion methods to identify true positive human plasma proteins with X!TANDEM and achieve a high observation frequency of those proteins with SEQUEST. The commonly used protocol to digest human plasma after reduction and alkylation results in thousands of peptides from the most abundant protein albumin with a concomitant decrease in the detection of peptides from other proteins. In contrast, digestion without reduction and alkylation resulted in increased observation of many proteins other than albumin. The combination of digestion methods resulted in the largest plasma proteome recorded to date in a single experiment and agrees with previous estimates of ≥ 12,000 plasma proteins from the results of multiple laboratories combined.^1−3,5^

### Peptide Observation Frequency

Peptide observation frequency is a powerful metric that can be used to make comparisons between experimental treatments^24,25,50^ or to compute the probability that the corrected observation count data are the same as random MS/MS spectra.^45,46^ Peptide observation frequency might be corrected by the counts per experiment, the sum of peptides identified, or the number of MS/MS spectra recorded.^26,27,45,46,51^ A high observation frequency is required to compare peptide observations between treatments with good confidence.^24,25,50^ Plasma protein isolation and digestion conditions that permit multiple peptide detections from a large number of proteins would be most useful for experimental analysis of plasma for the discovery of biomarkers, where the amount was quantified by observation frequency. The tendency of reduction and alkylation of serum samples to result in thousands of peptide observations from albumin, which comprises the bulk of plasma protein, may mask the observation of some other important proteins in the sample. Digestion of serum proteins with trypsin alone followed by DTT and trypsin for microflow electrospray to Paul ion trap yielded 4,396 distinct protein gene symbols from fully tryptic peptides without cysteine alkylation by the X!TANDEM algorithm.^11^ However, the use of nanospray peptide chromatography^52^ together with the sensitive^41^ linear quadrupole ion trap^53^ should result in much higher peptide observation frequency as observed.

### X!TANDEM versus SEQUEST

X!TANDEM^32^ is a very rigorous MS/MS fit algorithm that shows a low type I error and returns few results from noise or random MS/MS spectra but requires excellent quality MS/MS spectra as a substrate for successful identification.^45,46^ Higher observation frequencies were obtained from the sensitive SEQUEST algorithm that is perhaps the most popular and highly cited MS/MS correlation algorithm.^33^ The X!TANDEM and SEQUEST algorithms showed qualitative and quantitative agreement after correction and provided the complementary information of true positive protein identity from X!TANDEM and large protein observation frequency from SEQUEST.^7,24,25^ Correcting SEQUEST against noise and random MS/MS spectra together with imposing an XCorr cutoff score of ≥2 apparently controlled the error of SEQUEST and showed good agreement with the directly measured *p*-values obtained from X!TANDEM. The X!TANDEM algorithm has been repeatedly ground tested against negative control random MS/MS spectra and blank noise injections but also positive control protein standards and shown to be a reliable “gold standard” of protein identification from the fit of MS/MS spectra.^26,27,39,42,45,46,51,54^ The highly significant regression between X!TANDEM and SEQUEST on 9,935 protein gene symbols provides clear quantitative evidence of the veracity of fitting MS/MS spectra from heavy isotopes and hydrogen rearrangements in addition to the monoisotopic mass. The X!TANDEM FDR *q*-values and the SEQUEST observation frequency corrected against analytical and statistical controls showed that direct MS/MS algorithms have the selectivity and statistical power to discern true positive proteins of blood plasma from isotopic and other peptides.^35^

### Trypsin Digestion Conditions

The heterogeneity of peptides reported in plasma seems to indicate that LC-ESI-MS/MS is sensitive to differences in digestion conditions.^1^ Different digestion conditions might be exploited to identify a greater number of proteins from human plasma. The traditional approach of reduction, alkylation, and digestion resulted in many thousands of peptide observations from albumin. In contrast, trypsin alone resulted in a greater number of protein gene symbols with multiple independent peptide correlations, as expected. The presence of high abundance peptides from albumin may compete for ionization current, and the fraction of the machine cycle time spent sampling albumin peptides may preclude the detection and quantification of peptides from other less abundant proteins. Avoiding the highly redundant detection of albumin in the absence of reduction and alkylation may permit more sensitive detection of some other serum proteins. We conclude that trypsin digestion alone was the most efficient strategy to identify and count peptides and proteins. The detection of some cellular proteins from plasma significantly increased in the absence of reduction and alkylation, which might be important for biomarker discovery.

### Plasma Proteome

We have previously summarized the number of protein types reported using SQL versus BLAST to estimate the size of the human plasma proteome.^1,3−5,51^ The results here agree with an estimate of a set of at least 12,000 proteins in human plasma from mass spectrometry by the combination of multiple methods, instruments, laboratories, and approaches.^1−3^ In contrast, the random MS/MS spectra from the Monte Carlo control were randomly distributed across the database to all proteins but tend to accumulate in giant proteins like TTN, HLA(MHC), NEB, SYN, OBSC, MACF1, PLEC, and others that must be corrected.^7,26,27,36^ The highly significant and quantitative agreement between the monoisotopic and isotopic peptides from the OIT that showed excellent agreement with the corrected results of the LIT^41^ provides strong biophysical evidence for the veracity of the plasma proteome collected by the robust and sensitive LIT data after correction by noise and random spectra. Given the transposition driven evolution of proteins along with the possibility of alternative RNA splicing from the same gene,^55,56^ the use of protein gene symbols to summarize LC-ESI-MS/MS is a conservative estimate and has the added benefit that all genetic, biochemical, and genomic data are already organized under gene symbols.^57,58^

### Biological and Clinical Importance

The use of the rigorous X!TANDEM algorithm with analytical and statistical controls to define the set of true positive plasma proteins together with the independent estimation of observation frequency of those proteins across treatments by SEQUEST may be used for biological investigation.^24,25,7^ The strategy of trypsin digestion without reduction and alkylation has resulted in the discovery of the fetal growth factors from fetal verses adult serum that was confirmed by the exogenous addition of the discovered growth factors^25^ and immunological proteins that differ between fetal and adult serum that were confirmed by ELISA.^7^ Moreover, the same strategy of defining true positive plasma proteins with X!TANDEM and measuring relative observation frequency by SEQUEST after correction revealed the cellular biomarkers of COVID-19 and lung failure that were confirmed by enzyme assays.^24^ In contrast, the reduction and alkylation strategy only rediscovered existing acute phase markers like AZGP1, B2M, CRP, HP, HPR, ORM, RBP4, and SAA that may be repurposed as biomarkers of COVID-19.^59−69^ Similarly, autodigesting plasma using the weak endogenous trypsin activity^70^ without reduction and alkylation resulted in the identification of biomarkers of sample degradation,^36,71,72^ sepsis, ovarian, and breast cancer, and Alzheimer’s dementia that indicated the presence of ∼13,000 plasma proteins from X!TANDEM.^43,50,73−75^ Thus, there is no requirement for reduction and alkylation for biomarker discovery from plasma, and in fact, all available evidence indicates that digestion with trypsin without alkylation has successfully enumerated the biomarkers of blood fluids. Herein, the strategy of defining the true positive human plasma proteome by X!TANDEM with FDR corrected *q*-values and Monte Carlo correction against noise and random MS/MS spectra revealed the differences between tryptic digestion protocols with high confidence. Digestion with trypsin alone increased the observation frequency for a larger number of low abundance and cellular proteins from blood, and together, the complementary methods identified the largest X!TANDEM plasma proteome to date which is of great basic and clinical importance.

## Materials and Methods

### Materials

The HPLC instrument was Agilent 1100 (Santa Clara, CA, USA). Linear ion trap mass spectrometer LTQ XL was from Thermo Electron Corp (Waltham, MA, USA). Ceramic quaternary amine resin was from BioRad (Hercules, CA, USA). HPLC solvents were obtained from Caledon Laboratories (Georgetown, Ontario, Canada). The Dionex UltiMate 3000 series Ultra-Performance Liquid Chromatography (UPLC), C18 Acclaim PepMap Nano column (75 μm ID, 25 cm length, C18), Fusion Lumos Orbital ion trap, and UPLC solvents water and acetonitrile (ACN) were obtained from Thermo Fisher Scientific (Waltham, MA, USA). C18 ZipTips were obtained from Millipore (Bedford, MA). C18 HPLC resin was from Agilent (Princeton Sphere 300 C18, 5 μm, 300 Angstrom). Buffers and other reagents were from Sigma Aldrich (Saint Louis, MO USA). Trypsin was obtained from Promega (Madison, WI USA). The normal human plasma was from St Michaels Hospital (Unity Health), Toronto, Canada, REB# 20–078.

### Acetonitrile Precipitation

Plasma aliquots of normal human plasma (25 μL) were precipitated with 250 μL of room temperature 100% acetonitrile for 15 min and then centrifuged at 15,000 RCF for 5 min before removing the supernatant. The proteins were dried under vacuum for 30 min, and the freeze-dried plasma was stored at −80 °C for subsequent analysis.

### Quaternary Amine (QA) Protein Chromatography (SAX)

The proteins from 25 μL of serum were dissolved in 200 μL of 20 mM tris pH 8.85 binding buffer, fractionated over quaternary amine resin, and eluted with NaCl prior to tryptic digestion after the method of Tucholska.^11^ A new disposable preparative 100 μL quaternary amine chromatography column was created for each plasma sample.^11^ The quaternary amine column was fabricated in a 1.5 mL transfer pipet by cutting off the top of the bulb to act as a buffer reservoir and packing the tip with a glass wool frit before adding 200 μL of 50% resin slurry and permitting the column to pack under buffer within a 15 mL tube.^11,18^ The resin was equilibrated with a binding buffer prior to introducing the sample by gravity. The resin was washed with 3 volumes of buffer prior to elution with 300 mM (*N* = 4), or 600 mM (*N* = 15), or in a 50 to 1,000 mM 24 step gradient of NaCl (*N* = 3), where (*N* = 3 × 24 = 72) for each digestion treatment (4 + 15 + 72 = 91 LC-ESI-LC-MS/MS runs). To avoid cross contamination, the preparative quaternary amine column was discarded after a single use. The protein content of the fractions was measured by the dot blot protein assay.^76^

### Tryptic Digestion

The human plasma samples from quaternary amine chromatography were then digested in 20 mM tris pH 8.85 in 600 mM urea and 5% acetonitrile for ≥ 16 h under three different conditions: (1) trypsin 1/100 wt/wt;^18^ (2) reduced in 2 mM DTT for 30 min at 50 °C before digesting;^11^ or (3) reduced in 2 mM DTT for 30 min at 50 °C before adding 15 mM iodoacetamide and reaction in the dark for 1 h followed by quenching with 5 mM DTT and tryptic digestion with 1/100 trypsin (wt/wt).^21^ The digested samples were then quenched with 5% formic acid, dried under vacuum in a rotary lyophilizer, and stored at −80 °C for subsequent analysis.

### C18 ZipTip of Peptides

Preparative C18 column separation provided the best results for peptide analysis in a “blind sample” comparison.^77^ A new disposable C18 preparative “ZipTip” column was used to collect each digestion sample prior to injection. Solid-phase extraction with C18 for LC-ESI-MS/MS was performed as previously described.^18,78^

### Analytical HPLC LC-ESI-MS/MS

The tryptic peptides from normal human plasma samples were analyzed by LC-ESI-MS/MS. To prevent any possibility of cross contamination between treatments (trypsin vs DTT trypsin vs DDT alkylation trypsin), a new disposable nano analytical HPLC column and a nano emitter were fabricated for recording each set of protein fractions (3 treatment × three replicates = 9 columns). The mass spectrometers were cleaned, tuned, and tested for sensitivity with angiotensin and glufibrinogen prior to recordings.^26,27^ Each disposable C18 analytical column was conditioned and quality controlled with a mixture of three nonhuman protein standards using a digest of bovine cytochrome C, yeast alcohol dehydrogenase (ADH), and rabbit glycogen phosphorylase B to confirm the sensitivity and mass accuracy of the systems.^26,27^ The conditioned column was extensively washed in 50% acetonitrile before the sample fraction set. The peptides in 2 μL of eluted sample from ZipTip were diluted with 18 μL of 5% formic acid in water and immediately loaded manually into a 20 μL metal sample loop before introduction to the analytical column via a Rheodyne manual injector at a flow rate of ∼ 10 μL per minute. The peptide samples were analyzed over a gradient and split upstream of the injector during recording to ∼200 nL per minute. The analytical HPLC separation was performed with a Princeton Sphere 300 C18, 5 μm, 300 angstrom (150 mm × 0.15 mm) fritted capillary column. The acetonitrile profile started at 5%, ramped to 12% after 5 min and then increased to 65% over ∼90 min, remained at 65% for 5 min, decreased to 50% for 15 min, and then declined to a final proportion of 5% prior to injection of the next step fraction from the same serum sample.^11^ The nano HPLC effluent was analyzed by ESI ionization with detection by MS and fragmentation by MS/MS with a linear quadrupole ion trap.^53^ The device was set to collect the precursor for up to 200 ms prior to MS/MS fragmentation with up to four MS/MS fragmentations per precursor ion.

### Sampling Strategy

The separate results of the trypsin treatment, DTT trypsin treatment, and DTT alkylation and trypsin treatment from three independent experiments on three different dates were combined for statistical analysis. A total of 273 LC-ESI-MS/MS experiments were performed comparing trypsin (*N* = 91), DTT and trypsin (*N* = 91), and DTT and iodoacetamide plus trypsin (*N* = 91). The peptides from the three treatments were then randomly and independently sampled with respect to time as the peptides were eluted from the C18 chromatography column by nano LC-ESI-MS/MS.^36^ A total of 29 blank nano LC-ESI-MS/MS runs were performed where no sample was injected served as the baseline and statistical control alongside 30,000,000 random MS/MS spectra that served as a statistical control of observation frequency.^26,27^

### Orbital Ion Trap (OIT) Confirmation

Human normal plasma samples were prepared as above with trypsin alone and DDT followed by trypsin without alkylation as previously described.^11,18^ The plasma peptides were collected over C18 ZipTip in 5% acetic acid, washed and eluted in 2 μL of 5% formic acid and 65% acetonitrile, and immediately diluted with 18 μL of 5% formic acid for injections via a 20 μL loop. The resulting peptides were analyzed over an Acclaim PepMap Nano LC column (C18, 2 μm particles with a pore size of 100 Å, ID: 0.075 mm × 250 mm) at 30 nL per minute with a gradient from 5 to 70% acetonitrile over 60 min for Thermo orbital ion trap, Fusion Lumos (Q-Orbital ion trap-LTQ Trihybrid MS), at the National Taiwan University, Department of Chemistry. The electrospray voltage was 2000 V, and the ion transfer tube was at 275 °C. The tryptic peptides were identified by MS scans from 350 to 1700 *m*/*z* and followed by HCD-MS/MS of the most intense ions at a normalized collision energy of 32%. AGC target 5e4 was set for MS/MS analysis with previously selected ions dynamically excluded for 60 s with an isolation width of 1.4 Th. The maximum injection time was set to 50 ms. The resulting MS/MS spectra were correlated with the X!TANDEM and SEQUEST algorithms as described above but were also searched by the MaxQuant algorithm^79^ without modification, and SQL Server was used to remove the redundant use of MS/MS spectra by MaxQuant to prevent over interpretation of the results.

### Correlation Analysis

Correlation analysis of ion trap data was performed with the X!TANDEM and SEQUEST algorithms^32^ to match tandem mass spectra (MS/MS) to peptide sequences from the UNIPARC federated library of 157,636 known or hypothetical human protein accessions or loci that differed by at least one amino acid and contains all historical accession numbers and description fields.^36,75^ The MS/MS spectra from precursors greater than 1000 (≥e3) arbitrary counts from nano LC-ESI-MS/MS were fit to fully tryptic peptides with or without oxidation or other modifications. The statistical algorithms^2^ were used to directly fit MS/MS from precursor ions of 350 to 2000 *m*/*z* to fully tryptic peptides with the previously determined optimal wide mass error settings^26,27^ to account for isotopes and apparent hydrogen rearrangements^37,38,80^ without arbitrary heuristic methods. The SEQUEST and X!TANDEM algorithms were applied with a charge state of 2+ or 3+ with ± 3 *m*/*z* values for precursors and ± 0.5 Da for fragments with up to 3 missed cleavages for SEQUEST.^7−9,15,24,25^ The X!TANDEM algorithm computed the protein *p*-value with a maximal accepted value of *p* ≤ 0.01.^26,27^ The fully tryptic peptides [RK]|[X] were accepted with modifications such as glycine (G) substitution at cysteine (C) (57.021464) [carbamidomethyl, i.e., CAM modification at cysteine], the oxidation of methionine (M) or tryptophan (W) (15.994915), the addition of plus one at N and Q (0.984016), and sulfonation at M or oxidation of W (31.98983), as well as C-terminal addition of hydroxyl (17.002735) and N-terminal addition of a proton (1.007825). The results of the X!TANDEM and SEQUEST algorithms were stored together and compared in the SQL Server/R statistical system.^2,36,75^

### SQL Server Data Redundancy Error Elimination

The computational error from the redundant fitting of the same MS/MS spectra to more than one peptide sequence or modification was addressed by assigning a unique spectral ID number and using a complex key in SQL Server to accept the best fit per MS/MS spectra (BFPS) such that no MS/MS spectra could be assigned to more than one peptide in the X!TANDEM or SEQUEST peptide pools. The database of 29 blank LC-ESI-MS/MS runs of naïve columns^26,27,45,46^ was recorded to provide the blank frequency value for every peptide observed from experimental samples entering the database. Experimental peptide sequences with observation frequency similar to that of peptides observed noise MS/MS spectra from 29 blank runs from mock sample injections were discarded from the study (χ2 ≥ 9, *p* ≤ 0.01).

### Statistical Testing

The *p*-value from the exact fit of the MS/MS spectra to peptides and the cumulative protein gene symbol *p*-value were computed from X!TANDEM^32^ and corrected to a false discovery rate (FDR) *q*-value using the standard method of Benjamini and Hochberg to provide true positive results.^49^ SEQUEST may be corrected using random MS/MS spectra, filtered by a cross correlation score, (XCorr) and/or in agreement with X!TANDEM to yield true positive results.^7,18,24^ The corrected results from the X!TANDEM and SEQUEST peptides computed in R were previously validated against noise and random MS/MS spectra by chi-square distribution.^26,27^ Comparing experimental observation frequency to random proteins^81^ or random MS/MS spectra was previously used to control type I error rates.^26,27,45,46,51^ The observation frequency distribution of the 5,410,083 authentic plasma MS/MS spectra over protein accessions was compared to that of 30,000,000 random MS/MS spectra that showed the similar precursor and fragment number and mass distributions.^45,46^ Experimental peptide sequences with observation frequency similar to that of peptides observed from the 30,000,000 random MS/MS spectra controls were discarded from the study (χ2 ≥ 9, *p* ≤ 0.01).

## Supporting Information Available

The effect of treatments on the observation frequency of albumin and other plasma proteins is found in Tables S1–S4 and Figures S1–S3. The human plasma database showing the X!TANDEM observation count *n*, FDR *q*-value, and agreement with SEQUEST *n* over protein gene symbols is found in the file by Chen et al. (PDF)
Supplementary data (XLSX)

## Abbreviations

ACN: Acetonitrile
BFPS: best fit per MS/MS spectra
CAM: carbamidomethyl modification at cysteine
CBBR: Coomassie brilliant blue
DTT: dithiothreitol
IAA: iodoacetamide
HSA: human serum albumin
LC-ESI-MS/MS: liquid chromatography tandem mass spectrometry
LIT: linear quadrupole ion trap
Noise: blank LC-ESI-MS/MS injections
NHP: normal human plasma
OIT: orbital ion trap
QA: quaternary amine chromatography resin
TRYP: fully tryptic peptide search parameters
RSG: random MS/MS spectra generator
