# Supplementary material for: Trypsin Digestion Conditions of Human Plasma for Observation of Peptides and Proteins from Tandem Mass Spectrometry
Source: ACS Omega. 2024 Sep 24;9(40):41343–54. doi: 10.1021/acsomega.4c03955 (PMC11465567; doi:10.1021/acsomega.4c03955)
Supplement: Supplementary file 1 — ao4c03955_si_001.pdf [file ao4c03955_si_001.pdf]

Trypsin Digestion Conditions of Human Plasma for Observation of Peptides and Proteins from Tandem Mass Spectrometry

Zhuo Zhen Chen, Jaimie Dufresne, Peter Bowden, Ming Miao and John G. Marshall*

Research Analytical Biochemistry Laboratory, Department of Chemistry and Biology, Toronto Metropolitan University, Canada

SUPPLEMENTAL TABLES AND FIGURES

Supplemental Table 1. The effect of trypsin digestion conditions on the observation frequency of albumin and other plasma proteins by X!TANDEM versus SEQUEST. The sum of the three methods is also shown (Sum Methods T+DT+DAT). The number of best fit per MS/MS spectra (BFPS) peptides matched to each protein gene symbol after correction against noise and random MS/MS spectra are shown. The data is sorted by the sum of X!TANDEM T+DT+DAT in descending order. Treatments: Trypsin Alone (T); DDT reduction followed by trypsin (DT); DTT reduction, alkylation with iodoacetamide (IAA) followed by trypsin (DAT).

**Protein**	**Trypsin Alone (T)**	**Trypsin Alone (T)**	**DTT & Trypsin (DT)**	**DTT & Trypsin (DT)**	**DTT, ALK & Trypsin (DAT)**	**DTT, ALK & Trypsin (DAT)**	**SUM T+DT+DAT**	**SUM T+DT+DAT**
Gene Symbol	X!TANDEM	SEQUEST	X!TANDEM	SEQUEST	X!TANDEM	SEQUEST	X!TANDEM	SEQUEST
ALB	3787	12964	4700	106989	12402	83098	20889	203051
APOA1	3603	8001	1720	10380	1291	4232	6614	22613
SERPI01	1955	3498	942	4682	2732	3180	5584	11351
TF	1910	3489	776	4377	1497	1858	4154	10438
HP	1881	4203	755	5366	788	1297	3498	10161
C3	1756	2361	257	656	919	1114	2932	4131
A2M	1377	1728	511	2432	1332	3045	2858	6917
FGA	1238	1564	501	2434	1289	3021	2766	6854
APOA4	1153	2027	501	2431	1282	3008	2753	6814
HPR	1131	2732	126	281	831	629	2334	2638
HEL-214	1015	1440	271	1475	1302	1500	2192	3727
IGH@	976	1399	270	1473	1300	1503	2189	3726
IGHG1	970	1375	411	1924	609	1234	2151	5890
CP	883	1241	246	847	537	704	2021	3115
FGB	857	1300	288	1459	949	1525	1901	3888
HPX	833	1689	336	1573	571	1023	1628	4139
C4B_2	739	1239	245	1403	546	451	1624	3543
C4B	736	1237	145	598	576	360	1578	2258
C4A	728	1227	252	1548	836	1280	1560	3434
APOA2	721	1543	203	340	181	291	1532	2658
FGG	696	1138	278	1024	622	340	1456	2240
A1BG	684	1134	271	1019	584	336	1390	2219
IGHG4	664	904	181	703	722	402	1381	1992
HBB	629	962	267	688	640	342	1380	1667
IGHG3	619	750	267	688	639	342	1375	1667
IGL@	556	886	171	685	697	372	1273	1822
IgLC-rG	535	864	137	200	250	180	1270	1621
AHSG	509	516	193	822	371	279	1260	2239
HEL-213	479	892	221	776	516	274	1158	1816
IGK@	478	890	221	775	516	273	1158	1814
SNC73	473	637	106	261	295	346	1140	1846
IGHG2	472	606	106	259	280	333	1122	1829
IGHA1	469	637	107	254	280	332	1115	1813
ITIH2	461	756	199	230	212	162	1095	1526
IGLC2	421	766	204	209	215	393	1048	1564
IGLC3	421	766	205	465	398	274	954	1251
IGKC	405	765	138	703	455	259	879	1447
VTN	367	497	143	282	188	91	840	889
SERPI03	366	426	187	610	343	309	818	1596
AAT	363	597	152	617	342	203	767	1259
KNG1	358	650	119	648	394	168	718	1145
CFH	353	627	119	647	394	167	718	1144
ITIH1	352	476	185	2203	150	286	698	3086
IGHA2	351	512	129	556	201	520	697	1573
TTR	348	535	159	413	257	194	689	1046
KRT1	335	487	55	140	154	214	670	1110
CLU	321	515	130	234	177	145	660	1005
SERPING1	305	335	115	243	142	96	615	989

Supplemental Table 2. The effect of trypsin digestion conditions on the peptides observed per protein gene symbol. The sum of the T, DT and DAT methods is also shown (Sum T_DT_DAT). The peptides observed from plasma or cellular proteins without alkylation where DAT=0 and T_DT≥21 were selected and sorted alpha numerically. The number of best fit per MS/MS spectra (BFPS) peptides matched to each protein gene symbol after correction against noise and random MS/MS spectra are shown. Treatments: Trypsin Alone (T); DDT reduction followed by trypsin (DT); DTT reduction, alkylation with iodoacetamide (IAA) followed by trypsin (DAT).

GeneSymbol	Trypsin (T)	DTT_Trypsin (DT)	DTT_ALK_Trypsin (DAT)	Sum_T_DT_DAT	Search Engine
ACAN	62	46	0	108	SEQUEST
CPB2	59	8	0	67	X!TANDEM
FAM153A	42	14	0	56	SEQUEST
GPX3	45	3	0	48	X!TANDEM
pp14356	20	26	0	46	SEQUEST
ZC3H11A	28	17	0	45	SEQUEST
HBA1	28	15	0	43	X!TANDEM
PAPD5	37	6	0	43	X!TANDEM
APOC2	31	11	0	42	X!TANDEM
EPS8	21	19	0	40	SEQUEST
CLU	38	1	0	39	X!TANDEM
NEK4	25	14	0	39	SEQUEST
CD82	33	5	0	38	SEQUEST
AHCTF1	27	9	0	36	SEQUEST
USP28	28	6	0	34	SEQUEST
HBD/HBB	32	1	0	33	SEQUEST
ARNT2	25	7	0	32	SEQUEST
IRAK3	23	8	0	31	SEQUEST
ST3GAL1	23	8	0	31	SEQUEST
APOC1	26	4	0	30	X!TANDEM
HBA2	21	9	0	30	X!TANDEM
KIAA0319L	22	7	0	29	X!TANDEM
PKD1	22	7	0	29	X!TANDEM
ATRN	28	0	0	28	X!TANDEM
IFT122	24	3	0	27	SEQUEST
MIF	21	6	0	27	SEQUEST
NHLRC1	20	7	0	27	SEQUEST
DDHD2	24	2	0	26	SEQUEST
POLE1	21	5	0	26	SEQUEST
GP2	22	2	0	24	X!TANDEM
TTC13	24	0	0	24	X!TANDEM
APOM	23	0	0	23	X!TANDEM
ARMC7	21	1	0	22	SEQUEST
ARNT	20	2	0	22	SEQUEST
FBXL12	20	2	0	22	SEQUEST
IFT46	21	0	0	21	SEQUEST
PRM2	20	1	0	21	SEQUEST

Supplemental Table 3. The count of best fit peptides from MS/MS spectra matched to plasma proteins from digestion with trypsin alone (T), DDT and trypsin (DT) or DTT Iodoacetamide (DAT). The count of peptides with or without glycine (carbamidomethyl, CAM) modification at cysteine (C) (57.02) are shown.

Gene Symbol	CAM DAT	DAT	CAM_DT	DT	CAM_T	T
APOA1		247		423		967
ALB	7293	929	104	950	359	494
TF	1363	207	118	184	27	486
C3	108	76		51	3	348
A2M	116	75		22	3	277
HP	749	75	3	124	39	267
FGB	188	49		34	3	214
HPX	320	41	8	59	10	211
APOA4		22		47		205
HPR	209	39	4	64	18	127
APOA2	219	33	3	47	3	98
AAT		7		18		89
ITIH2		12		10		76
SNC73	254	48		52	8	74
APOC3		9		35		71
ITIH1		4		8	1	58
C1	201	21		18	6	40
TARS		92	1	87	2	40
SERPI3		3		3		35
HBA1				15		34
HBA2				14		33
SAA2-SAA4				10		31
SAA4				9		31
GC	123	20		27	3	28
HBD		6		12		22
ZDHHC14		13		19	1	22
LRG1				4		21
NUP153		17	2	12	1	21
HMFT1766				4		20
CARD10		1		4		19
APOC4-APOC2		1		11		18
APOC2		2		9		16
OK/KNS-cl.7		119		7		16
RPS2		119		7		16
RNF13		30		57		15
SDNK1		2			1	14
ND1		9		10	1	13
FOXF1		12		9		12
HIF-1A		10		3		11
LINC01118		7		4		11
PPP1R10		6		5		11
COL4A1		9		5	1	10
HSPD1		15		11		10
PCDH1		2		2		10
XTP11		2		5		10
APOH	213	2	6		5	5
HOXB3	114		24		36	1

Supplemental Table 4. Agreement of the sum of XTANDEM analysis of digestion treatments (T_DT_DAT) from the LIT versus OIT instruments searched by X!TANDEM and MaxQuant. Treatments: The sum of all digestion treatments from LIT linear ion trap searched by X!TANDEM, OIT monoisotopic data ± 0.1 from X!TANDEM, All precursor mass data from the OIT from X!TANDEM, and the OIT monoisotopic results ±0.1 Da from MaxQuant. Non-redundant peptide counts (N) per gene symbol are shown.

Protein Gene Symbol	X!TANDEM LIT _T_DT_DAT_N	X!TANDEM OIT _monoisotopic_N	XTANDEM_ All_OIT_N	MaxQuant Monoisotopic N
ALB	19616	642	2743	3336
APOA1	5920	270	641	685
TF	4968	265	764	1
HP	3618	166	515	413
IGH@	3419	228	824	8
SERPINA1	3021	228	583	713
HEL-214	2581	167	626	445
C3	2474	393	936	1022
A2M	1953	152	366	316
HPR	1850	103	214	45
FLJ00385	1818	110	375	25
FGA	1731	277	641	617
IGHG4	1705	95	368	26
IGL@	1637	145	432	115
DKFZp686C15213	1606	138	571	140
APOA2	1517	57	138	117
HPX	1418	126	323	263
APOA4	1345	196	425	558
IGK@	1312	118	428	420
FGG	1121	147	337	254
CP	1087	282	646	656
A1BG	967	99	266	256
HBB	942	95	252	219
GC	721	97	279	291
IGLC7	657	61	193	34
VTN	602	33	95	92
ITIH2	581	83	181	247
CFH	547	72	204	186
KNG1	532	72	157	158
TTR	473	104	232	310
CFB	389	76	146	162
HBD	381	55	138	14
SERPING1	369	26	60	34
APOC3	366	24	68	51
CLU	339	82	164	144
ITIH4	330	170	365	355
HBA2	309	65	151	189
ORM2	298	118	406	439
IGHM	291	64	143	125
SERPINC1	288	121	257	260
PLG	281	32	83	71
RBP4	249	35	113	132
GSN	205	59	138	163
V_lambda_3	187	20	47	8
CFHR1	177	12	39	3
FN1	171	23	58	45
C9	170	58	119	124
APOD	159	30	70	57

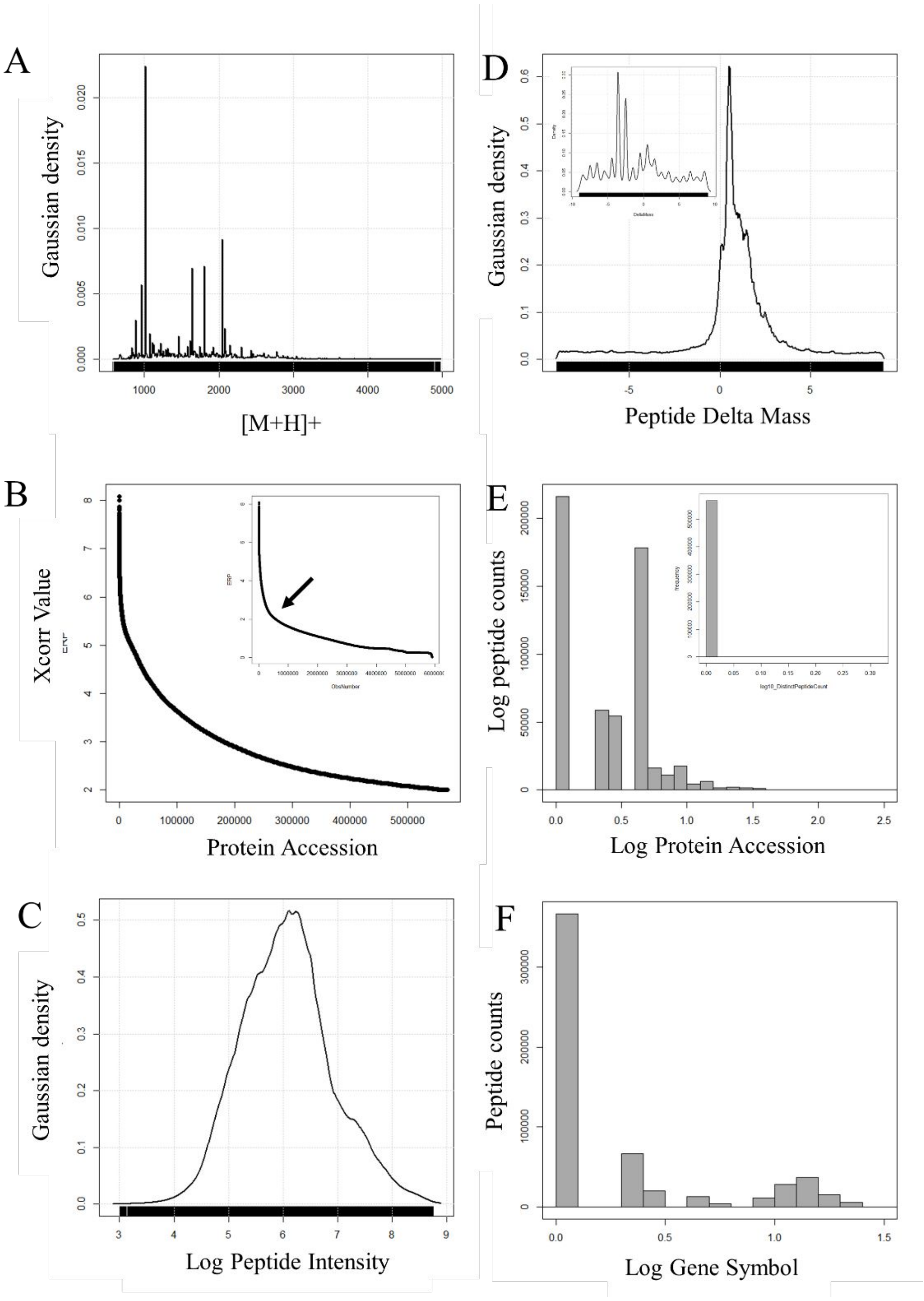

Supplemental Figure 1. The summary of human plasma at the level of protein accession numbers from the sum of SEQUEST from all three digestion conditions of trypsin. Panels: A, the MH distribution of peptides in Daltons; B, the peptide X-Corr distribution ≥ 2 (inset all XCorr values where arrow shows inflection at XCorr≥2); C, the peptide log precursor intensity distribution; D, the peptide delta mass distribution (observed-expected mass) in Daltons (Da) [SEQUEST inset delta mass distribution prior to correction]; E, the log peptide to protein accession distribution (inset the log MS/MS spectra to peptide distribution); F, the log protein accession to gene symbol distribution.

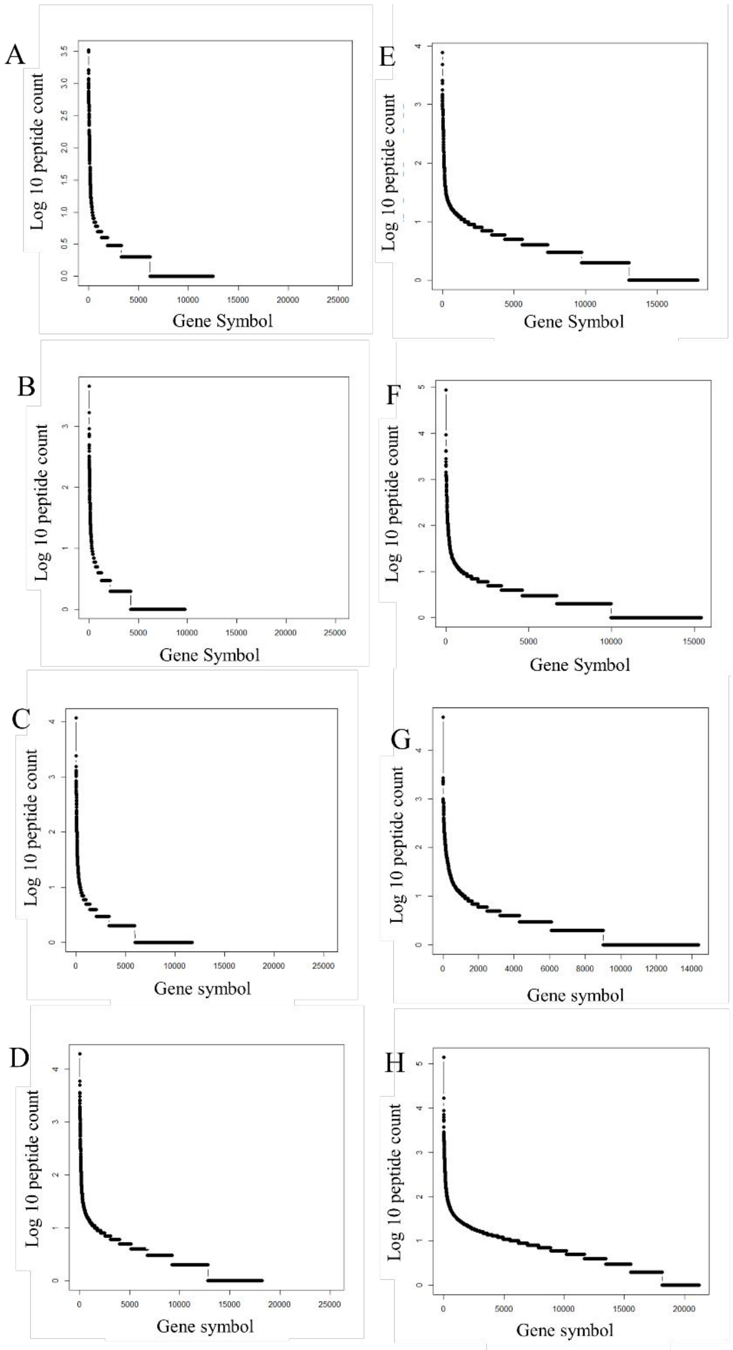

Supplemental Figure 2. The distribution of the peptide observation counts from noise and random corrected X!TANDEM results (left) and noise and random corrected SEQUEST results (right). Panels: A, the X!TANDEM log distribution of the peptides per gene symbol for trypsin alone; B, the X!TANDEM log distribution of the peptides per gene symbol for DTT trypsin; C, the X!TANDEM log distribution of the peptides per gene symbol for DTT IAA trypsin; D, the X!TANDEM log distribution of the peptides per gene symbol for the sum of all treatment; E, the SEQUEST log distribution of the peptides per gene symbol for trypsin alone; F, the SEQUEST log distribution of the peptides per gene symbol for DTT trypsin; G, the SEQUEST log distribution of the peptides per gene symbol for DTT IAA trypsin; H, the SEQUEST log distribution of the peptides per gene symbol for the sum of all treatments.

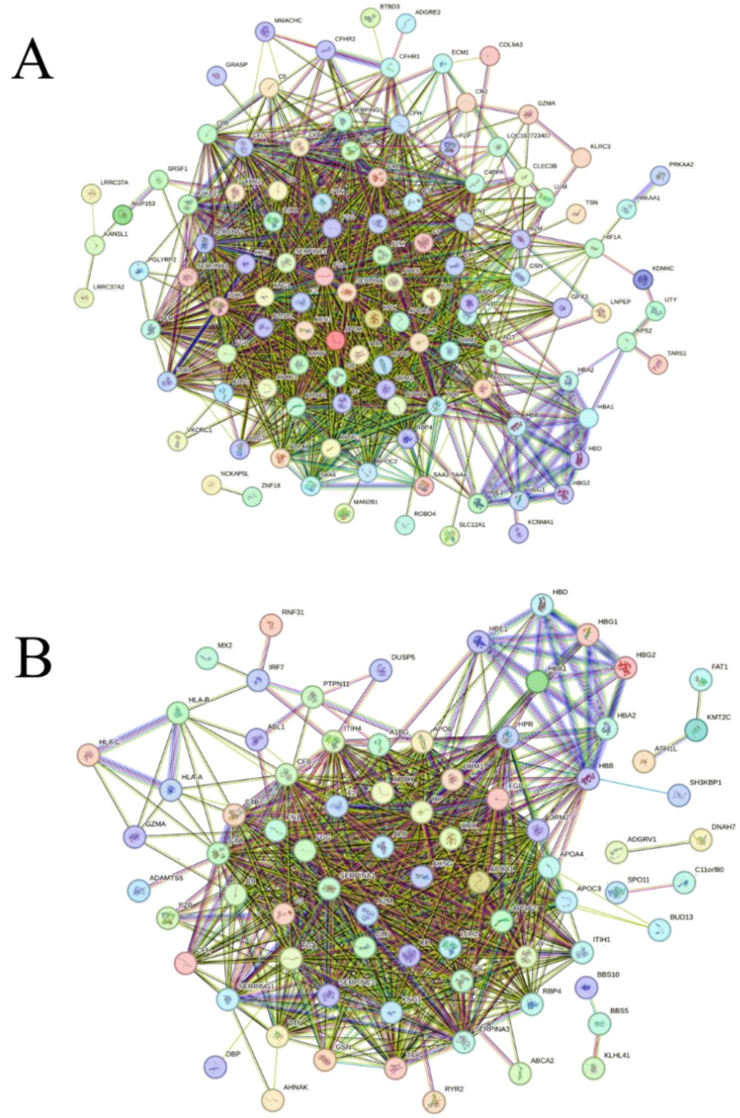

Supplemental Figure 3. The agreement on plasma proteins from corrected X!TANDEM versus SEQUEST from the sum of the different digestion conditions. Panels: A, gene symbols from X!TANDEM with 100 or more peptides [number of nodes: 88, number of edges: 1232, average node degree:28, avg. local clustering coefficient: 0.692, expected number of edges:76, PPI enrichment p-value:< 1.0e-16]; B, gene symbols from SEQUEST with 400 or more peptides [number of nodes: 139; number of edges:185, average node degree:2.66, avg. local clustering coefficient:0.362; expected number of edges:123, PPI enrichment p-value:1.03e-07]. The agreement set of more than 12,000 gene symbols is in the supplemental data with the corrected observation frequency of the plasma proteins with X!TANDEM gene symbol p-values and FDR q-values alongside SEQUEST where XCorr ≥ 2.
